# Profiling of SARS-CoV-2 virus shedding, antibody neutralization, and T-cell receptor repertoires in a large, multi-center cohort of young adults with varied prior exposures

**DOI:** 10.3389/fimmu.2026.1731974

**Published:** 2026-03-25

**Authors:** Andrew Fiore-Gartland, Koshlan Mayer-Blackwell, James Stray, Moni Neradilek, April Lo, Alex Hannah, Tracy Dong, Leonid Serebryannyy, Robin Carroll, Bob C. Lin, Richard A. Koup, Nina Marie G. Garcia, Jasmine R. Marcelin, Audrey E. Pettifor, Holly Janes, Elizabeth R. Brown, Catherine Yen, Jessica Andriesen, Lawrence Corey, Kathryn E. Stephenson, James G. Kublin

**Affiliations:** 1 Vaccine and Infectious Disease Division, Fred Hutchinson Cancer Center, Seattle, WA, United States; 2 Adaptive Biotechnologies, Seattle, WA, United States; 3 Vaccine Research Center, National Institute of Allergy and Infectious Diseases, National Institutes of Health, Bethesda, MD, United States; 4 Division of Infectious Diseases, University of Nebraska Medical Center, Omaha, NE, United States; 5 Department of Epidemiology, University of North Carolina at Chapel Hill, Chapel Hill, NC, United States; 6 Division of AIDS, National Institute of Allergy and Infectious Diseases, National Institutes of Health, Bethesda, MD, United States; 7 Center for Virology and Vaccine Research, Beth Israel Deaconess Medical Center, Harvard Medical School, Boston, MA, United States; 8 Ragon Institute of Mass General Brigham (MGH), Massachusetts Institute of Technology (MIT) and Harvard, Cambridge, MA, United States

**Keywords:** antibody neutralization, hybrid immunity, SARS-CoV-2, T cell receptor repertoire, vaccines, viral shedding

## Abstract

**Background:**

The cellular and neutralizing antibody responses to SARS-CoV-2 infection are complex, particularly with multiple and heterogeneous exposures. We sought to understand the changes to the antibody and T-cell repertoire elicited by mildly symptomatic or asymptomatic infections in young adults, and how immune responses to prior vaccination may interact with new viral exposures in this population. Additionally, profiling both aspects of humoral and cellular immunity from a single vial of blood is experimentally challenging.

**Methods:**

We developed a protocol to recover T-cell receptor (TCR) repertoires from frozen blood clots, i.e., remnant material, retained after coagulation of whole blood for serum recovery and antibody analysis. The method was applied to a subset of participants in a COVID-19 vaccine trial (CoVPN 3006, *n* = 209 participants). We sequenced TCR repertoires and measured anti-SARS-CoV-2 antibody responses from pre-exposure, post-vaccination, and post-breakthrough blood samples.

**Results:**

Clot material provided suitable genomic DNA for TCR profiling, with vaccination and infection leading to expansions in T-cell responses. Consistent with prior studies, we found that hybrid immunological exposures (vaccination after infection) lead to the greatest antibody potency and spike TCR breadth. When the order of exposure was reversed, we observed evidence of attenuated disease severity (reduced shedding duration and lower peak nasal viral load) in post-vaccination versus primary infections.

**Discussion:**

The protocols described here for recovery of TCR repertoires from remnant coagulated material will facilitate more common estimation of cellular and neutralizing antibody immune responses as potential correlates of protection in large clinical trial cohorts where peripheral blood mononuclear cell (PBMC) acquisition or analysis is otherwise infeasible.

## 1 Introduction

Many studies early in the COVID pandemic extensively characterized the humoral and cellular immune responses that remain after a SARS-CoV-2 infection and their association with the severity of disease (1, 2). With the advent of effective SARS-CoV-2 vaccines, later studies examined potential differences between immunity acquired from vaccination versus hybrid exposures (infection and vaccination) (3–7). However, because of the challenges associated with frequent longitudinal sampling of individuals prior to and near the time of infection, much less is known about the immune responses elicited by mildly symptomatic or asymptomatic infections in young adults, and how immune responses to prior vaccination may interact with new viral exposures in this population. As part of CoVPN 3006 (ClinicalTrials.gov NCT04811664), launched in March 2021, daily nasal swabs were collected prospectively and tested for SARS-CoV-2 by polymerase chain reaction (PCR) (8). This allowed for precise identification of mild and asymptomatic SARS-CoV-2 infections in a cohort with no severe disease, relative to the timing of randomized two-dose mRNA vaccination.

Identifying mechanisms of immunological control of SARS-CoV-2 and other viral infections requires standardizable measures of both neutralizing antibody and cellular responses to vaccination in participants across multiple clinical sites. Archived serum, the soluble cell-free supernatant routinely collected from whole blood coagulation and centrifugation, can be readily used for measurement of antibodies; however, cellular immunity is typically evaluated using live-cell assays, which present several challenges. First, storage of viable cells requires rapid sample processing, cryopreservation, and continuous cold-chain storage at −80°C. Variation in sample collection, handling, or storage protocols can result in reduced cell viability and inconsistent results, increasing costs and decreasing feasibility of measuring cellular immunity. Thus, in many large clinical trials (including CoVPN 3006), peripheral blood mononuclear cells (PBMCs) are not routinely collected alongside serum. To address this, we developed a protocol to co-analyze neutralizing antibody and cellular immunogenicity from a single blood sample by recovering genomic DNA (gDNA) for T-cell receptor (TCR) amplicon sequencing from T cells trapped in the recalcitrant pelleted fibrin meshwork composed of red blood cells (RBCs), leukocytes, and platelets (i.e., “blood clot” material).

Here, we present the analysis of a subsample from the CoVPN 3006 cohort (*n* = 209) in which we applied a novel method to simultaneously analyze the neutralizing antibody and cellular responses to SARS-CoV-2 exposure (infection or vaccination). Our findings suggest that using remnant cells in clot material saved from serum separator tube (SST)-collected blood is a reliable and reproducible method to profile TCRs. We show that the mechanism of exposure to SARS-CoV-2 and the order in which the exposures occur influence the immune response, such that vaccination after infection results in the greatest antibody potency and increased spike TCR breadth.

## 2 Materials and methods

### 2.1 Study population

We conducted a study of the immunogenicity and viral shedding of participants in a clinical trial that evaluated two doses of the mRNA vaccine mRNA-1273 (Moderna) for prevention of SARS-CoV-2 infection (CoVPN 3006 ClinicalTrials.gov NCT04811664); the cohort was enrolled from across the United States and details have been previously published (8). Briefly, adults aged 18–26 years were eligible if they were enrolled in a higher education institution, as the social living situation of university students made them more susceptible to SARS-CoV-2 infection and transmission than others in the age group, and participants randomized to the standard of care (SoC) arm were to be vaccinated at the end of the 4-month study. In May 2021, federal vaccine recommendations expanded to include all adults aged ≥18 years, reducing the pool of eligible unvaccinated young adults (9). Thus, in June 2021, the protocol was amended: the upper age limit at enrollment was raised from 26 to 29 years, and the requirement to recruit only students was removed. A vaccine-declined observational group was also recruited from 16 June to 8 November 2021 and followed up for SARS-CoV-2 infection as specified for the randomized participants. A central institutional review board approved the protocol and consent forms and subsequent amendments, and local institutional review boards approved site-specific consent forms and documents as appropriate. Participants provided written informed consent before enrollment. Please refer to the main study publication for additional details (8).

The CoVPN 3006 study was conducted from March 2021 to April 2022 at 30 sites in the United States (8). Participants for the randomized arms were assigned to immediate vaccination (immediate arm; e.g., receipt of two doses of mRNA-1273 vaccine at months 0 and 1) or SoC (e.g., receipt of COVID-19 vaccine per federal, state, and local guidelines or two doses of mRNA-1273 provided by the study team at month 4 and 28 days later at month 5, if vaccine was not received previously off study) in a 1:1 ratio using a centralized interactive response technology system. To assess infection via RT-PCR, participants were instructed to perform self-swabs on the anterior nares daily starting at vaccination or day 1 (depending on group) and continuing for 16 weeks and to return swab samples to sites three times per week. In the event of SARS-CoV-2 infection, participants provided an additional serum sample for SARS-CoV-2 clinical serology and performed daily eDiary symptom tracking for 28 days. Blood samples were collected at enrollment and longitudinally throughout the study.

### 2.2 Participant selection

For this study, participants were selected with serum samples collected proximal to exposure events. This included (i) persons with samples collected before and after an infection without prior vaccination, (ii) persons with samples collected before and after a vaccination, and (iii) persons with samples before and after a breakthrough infection occurring 10 or more days following a vaccination. Among vaccinated persons, a subset had serostatus measured for anti-SARS-CoV-2 nucleocapsid antibodies (*n* = 22 seropositive; *n* = 77 seronegative). Participants seropositive at the time of immunization acquired hybrid immunity following vaccination.

### 2.3 Sample stratification

Samples were stratified into groups based on time and nature of last exposure to SARS-CoV-2 antigens, whether via infection or vaccination. Samples collected within immunologically relevant duration were designated into categories distinguishing type and order of immunological exposures: I (infection, *n* = 27), V (vaccination-only, *n* = 75), H (hybrid exposure, *n* = 19) resulting from prior infection (anti-N seropositivity at time of vaccination) followed by vaccination, or VPstB (breakthrough infection) following two-dose vaccination (*n* = 12). Samples were considered if they were collected more than 10 days after the most recent exposures and prior to 45 days. Other samples collected within 45–90 days after the last known immunological exposure were categorized as V45, H45, and I45, respectively. Samples were designated seropositive (SP) if they came from individuals with anti-N seropositive status but were collected prior to PCR-confirmed SARS-CoV-2 infection or mRNA vaccination recorded in the study period. Samples from individuals with no known prior exposure were categorized as SN (seronegative, *n* = 77). Samples collected more than 135 days after the last recorded immunological exposure were excluded from analysis.

### 2.4 Daily PCR screening

As described previously (8), infection was assessed by detection of viral RNA in nasal swab samples. Participants were instructed to perform self-swabs on the anterior nares daily starting at vaccination or day 1 (depending on group) and continuing for 16 weeks and to return swab samples to sites three times per week. Testing with reverse-transcription PCR [SCV2-SPX-EP Molecular Test (Corteva Agriscience) or LabGold ultra-high-throughput SARS-CoV-2 end-point PCR (Northwell Health Laboratories)] was performed on every-other-day specimens, and a positive PCR result triggered testing of swab samples from 3 days before to 14 days after the positive specimen or until viral RNA was no longer detected. Positive PCR results were reported back to the site and participant. The infecting variant was determined by Spike sequencing of the peak viral load swab sample using Illumina NextSeq 500 and Illumina NextSeq 2000 whole-genome sequencing. Viral load was inferred as inversely proportional to qPCR critical threshold (Ct). Shedding duration was based on number of days with qPCR Ct ≤ 40.

### 2.5 Anti-N antibody assay

As described in the main study, participants were assessed for SARS-CoV-2-binding antibodies specific to the SARS-CoV-2 nucleocapsid protein [Roche Elecsys Anti-SARS-CoV-2 Immunoassay (University of Washington Retrovirology Laboratory, Seattle)] at weeks 0, 8, and 16. COVID-19 symptom surveillance was performed weekly by means of eDiary. In the event of SARS-CoV-2 infection, participants provided an additional serum sample for SARS-CoV-2 clinical serology and performed daily eDiary symptom tracking for 28 days. Lifestyle circumstance questionnaires pertinent to potential SARS-CoV-2 exposure were administered at baseline and weekly thereafter.

### 2.6 Pseudoneutralization assay

Samples were heat-inactivated at 56°C for 60 min. Heat-inactivated samples were assayed for SARS-CoV-2 (D614G, Delta, BA.1, BA.5, and BQ.1.1) neutralization with a lentiviral pseudovirus-based assay and reported as reciprocal 50% inhibitory dilution (ID_50_) or 80% inhibitory dilution (ID_80_) with a value of 20 (minimum dilution tested) plotted for samples that did not neutralize, as previously described (10).

### 2.7 Recovery and dissolution of remnant clotted blood from frozen stored post-spin SSTs

Blood was collected in SSTs (BD Vacutainer^®^ SST™), and the serum was separated from clotted blood by centrifugation per the manufacturer’s recommendations (1,000–1,300 × *g*/10 min) and used subsequently for serological analysis of study participants. Remnant clotted blood was frozen at −80°C while still in the SST, transported, and stored frozen, and thawed just prior to clot recovery. Rescue of clotted blood material was accomplished by placing SSTs into 50-mL conical tubes in an inverted orientation and spinning without refrigeration at 2,500 × *g* in a swinging bucket rotor (Thermo Fisher Multifuge X1 Pro with TX-1000 rotor) for 5 min. This simple procedure allowed clots to move past the separator plugs, coming to rest in the bottom of each conical tube, and for the spent blood tubes to be removed, leaving clot material behind. Clots were dissolved (liquified) by digestion with 750 µL of Proteinase K (MagMAX Ultra-Sample Ultra 2.0 Proteinase K) with vigorous mixing at 1,200 rpm for 10 min on an Ohaus High Speed Orbital Shaker (model #SHHSMPDG) at room temperature. To stabilize samples during orbital shaking, 50-mL conical tubes were placed in foam adapters (VWR 20 to 25 mm Tube Holder model #58816) and clamped between the lower platform and upper hold-down plate. Clots were completely or nearly completely dissolved following this treatment, and any remaining undigested material was typically small and could be pipetted through 1-mL standard pipette tips with minor teasing. The recovered liquified blood clots represent blood concentrates that, while enriched in RBCs, platelets, and leukocytes, provide a rich source of amplifiable gDNA with a genomic composition very similar, if not identical, to unfractionated peripheral whole blood. Prior to DNA extraction, clot lysates were centrifuged at maximum speed (3,800 RPM/3350 × *g*) to pellet insoluble material and bring down droplets that accumulated on the sides and lid during shaking incubation. Liquified clots can be processed immediately or stored frozen at −80°C for DNA extraction later without affecting sample integrity (data not shown).

### 2.8 DNA extraction

Leukocyte gDNA was purified from dissolved blood clots using a modification of the MagMAX™ DNA Multi-Sample Ultra 2.0 kit (Applied Biosystems/Thermo, Cat#A36570) automated on the KingFisher Flex™ Magnetic Particle Processor fitted with a 24-position magnet, operating with KingFisher 24-deep-well plates. To begin, lysis plates were set up by adding 1.5 mL of liquified clot material to wells containing 500 μL of molecular biology grade water and 200 μL of MagMAX DNA Ultra 2.0 Proteinase K. Next, 200 μL of MagMAX DNA Ultra 2.0 Enhancer Solution was added to the clot/water/ProK mixture, and the plate was sealed, placed on an Eppendorf ThermoMixer C, and mixed at 400 rpm for 3 min. Next, the seals were removed and the lysis plate was loaded on the KingFisher Flex, along with additional plates filled with other kit reagents per kit instructions (pages 7–8, Pub. No. MAN0017325 E00) for the KingFisher 2-mL whole blood extraction automated protocol (MagMAX_Ultra2_2mL_V2_Flex.bdz), eluting in 300 µL of MaxMAX DNA Ultra 2.0 Elution Solution. DNA was quantified by ultraviolet (UV) absorbance, 260 nm, and stored frozen prior to NGS TCR beta repertoire profiling by the Adaptive Biotechnologies immunoSEQ hsTCRB assay.

### 2.9 Peripheral blood collected in SST versus K2-EDTA vacutainer tubes

To demonstrate concordance between TCRB repertoire profiles for gDNA purified from remnant frozen clotted blood and whole, unclotted blood collected in K2-EDTA tubes, peripheral blood was drawn into each tube type from four healthy volunteer donors at a BloodWorks Northwest collection center. Blood was delivered to the Adaptive ISO 13485 certified R&D laboratory the day of the blood draw and SST clotted blood samples were fractionated by centrifugation at 1,300 × *g* for 10 min without refrigeration and the serum was decanted. These serum-depleted SST tubes containing remnant clotted blood were then frozen at −80°C to simulate stored SARS-CoV-2 test samples. To serve as an unclotted control, K2-EDTA whole blood tubes were frozen at −80°C and thawed, and DNA extractions were performed as described above, except that 2.0 mL of neat thawed blood was added to the lysis plate. An equivalent mass of 18 µg of gDNA from SST blood clots and K2-EDTA whole blood was analyzed by the TCRB immunoSEQ assay (**Figures 3G–I**).

### 2.10 T-cell receptor variable beta chain sequencing

Immunosequencing of the CDR3 regions of human TCRβ chains was performed (Adaptive Biotechnologies, Seattle, WA). Extracted gDNA was amplified in a bias-controlled multiplex PCR, followed by high-throughput sequencing. Sequences were collapsed and filtered to identify and quantitate the absolute abundance of each unique TCRβ CDR3 region for further analysis as previously described (11–13). The fraction of T cells was calculated by normalizing TCRβ template count to the total number of cells sequenced on the assay using a panel of reference genes found in all nucleated cells.

### 2.11 Mapping of SARS-CoV-2 TCRβ sequences

Peripheral repertoires were mapped against a set of TCR sequences that were known to react to SARS-CoV-2. Briefly, these sequences were first identified by Multiplex Identification of T-cell Receptor Antigen Specificity (MIRA) (14). SARS-CoV-2-specific TCR sequences identified via MIRA correspond to antigen-specific addresses, which detail MHC class presentation and were used to correlate response with viral ORF and cellular subsets (CD8 versus CD4). Reactive TCRs were further screened for enrichment in a previously SARS-CoV-2-infected repertoire compared to SARS-CoV-2-negative repertoires collected as part of ImmuneCODE, a publicly available open database, to remove TCRs that may be very frequent or cross-reactive to common antigens. The filtered list represents a set of TCRs that are both experimentally observed expanding to SARS-CoV-2 antigens as well as enriched in COVID-19-infected participants (15, 16). Responses were quantified by the number and/or frequency of SARS-CoV-2 specific TCRs. Specifically, clonal breadth (defined as the proportion of distinct TCRs that are SARS-CoV-2 specific divided by the number of unique TCRs sequenced in the sample) and clonal depth (defined as the sum frequency of the SARS-CoV-2-specific TCRs in the repertoire) were determined for each sample. These two metrics have been shown to have good separation of cases (defined as either COVID-19 infected or vaccinated) and controls previously (14–16).

### 2.12 Viral load and viral shedding analysis

Peak viral loads and viral shedding durations were estimated using a Bayesian hierarchical model fitted to viral load and seroconversion data from participants who had at least two positive qualitative qPCR samples and at least one positive quantitative qPCR sample. An extension to the methods described in Dong and Brown (17), the model assumed a piece-wise linear latent viral shedding trajectory for each person and took all the observed cycle threshold (Ct) values for the N1 and N2 targets, antibody tests results, and selected baseline covariates including male sex, whether the viral shedding started after 0, 1, or 2 doses of COVID-19 vaccination, and probability of Delta variant shedding (based on estimates from GISAID, the Global Initiative on Sharing All Influenza Data) as input (18), and produced posterior samples of estimates for peak viral load on the log10 copies/mL scale above the level of detection (LoD) and total number of days of viral shedding. Informative priors were used to estimate the intercept and coefficient when converting Ct values to log viral load based on prior literature (19). We ran two independent chains with 20,000 burn-ins and 20,000 additional samples with 10-step thinning, resulting in 4,000 posterior samples. Computations were carried out using R version 4.4.2 and JAGS 4.3.1. For participants with only one positive qualitative sample and one positive quantitative sample, we set the total shedding duration to be 1 day and use the model-estimated conversion formula to convert their observed minimum Ct to log10 copies/mL as their imputed peak viral load above the LoD. As a sensitivity analysis, we also estimated peak viral load as the crude minimum Ct value for each participant across all swabs.

### 2.13 Statistical comparisons

Sample groups were combined using non-paired non-parametric Wilcoxon rank-sum tests. For longitudinal comparisons before and after breakthrough infection, a paired Wilcoxon rank-sum test was used. Statistical analysis was performed in R version 4.3.3.

## 3 Results

### 3.1 Participants were sampled based on SARS-CoV-2 exposure

Briefly, the CoVPN 3006 study was conducted from March 2021 to April 2022 at 30 sites in the United States (8). Participants for the randomized arms were assigned to immediate or SoC and were instructed to perform self-swabs daily. Blood samples were collected at enrollment and longitudinally throughout the study. Participant demographics are shown in **Supplementary Table 1** and details are provided in Materials and Methods and in the main study (8).

**Figure 1:**
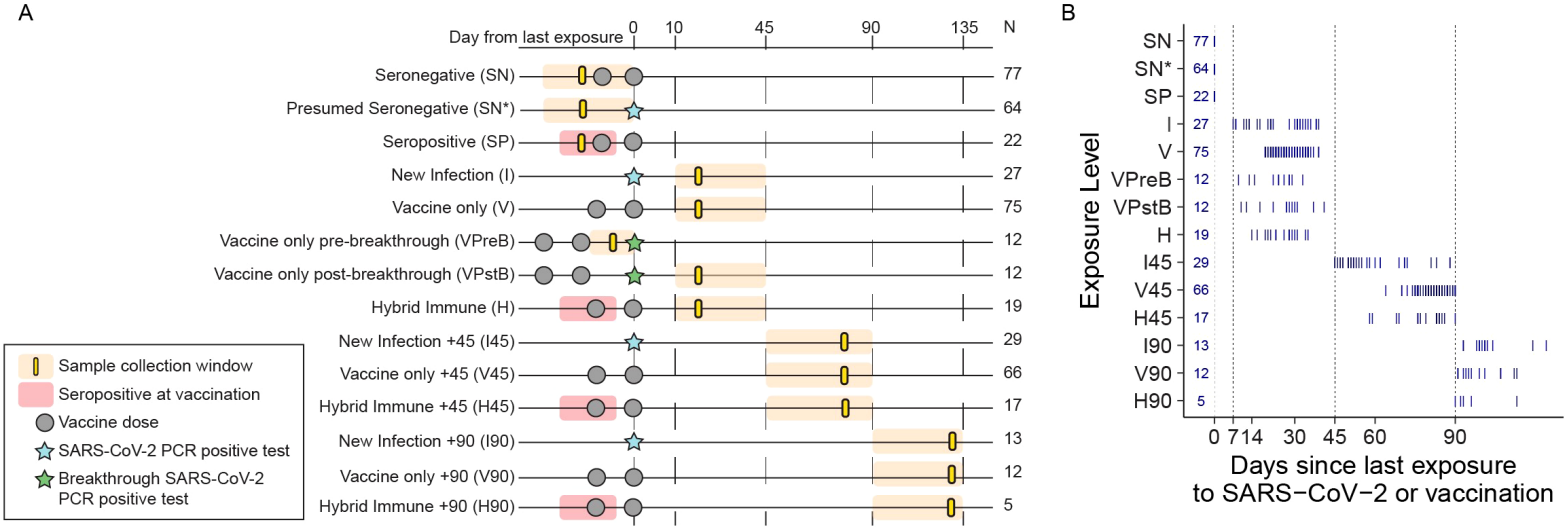
Samples were categorized relative to SARS-CoV-2 exposure events. **(A)** Schematic of sample categorization by timing from last exposure. **(B)** Days since last exposure by category. See Methods for sample stratification criteria.

Inclusion into this secondary analysis required a sample collected up to 90 days prior to exposure, or within 7–45 or 45–90 days after a vaccination or PCR-confirmed SARS-CoV-2 infection. In total, 450 samples met the inclusion criteria for this secondary analysis based on proximity to a PCR-confirmed SARS-CoV-2 infection or mRNA vaccination (**Figure 1A**). Among the participants with samples available, the majority of infections occurred prior to vaccination (*n* = 46 of 63), while 12 PCR-confirmed SARS-CoV-2 infections occurred from 7 to 90 days (median 49 days) after the second vaccination dose, now referred to as “breakthrough” infections. Participants provided nasal swabs throughout their participation in the study, with a mean of two swab samples per week throughout follow-up for all participants (8).

We analyzed immunological responses to two doses of mRNA SARS-CoV-2 spike vaccination (Moderna mRNA-1273) and/or SARS-CoV-2 infection in 209 participants (77 male and 132 female participants) ranging in age from 18 to 29 years old (median 21). At that time in the United States, Alpha and Delta SARS-CoV-2 variants were the most prevalent, consistent with viral genotyping of infections in the study that showed that more than 65% of infections were by Delta or Delta sub-lineages of the SARS-CoV-2 virus.

To facilitate comparisons of immunological responses following vaccination and/or infection in this secondary analysis, we assigned samples to groups based on the relative timing of the individual’s last known exposure (i.e., PCR-confirmed infection or completion of two-dose mRNA vaccination) (**Figure 1A**). Samples from individuals that were seronegative for SARS-CoV-2 nucleocapsid prior to vaccination were designated SN (*n* = 77) and those from presumed seronegative participants were designated “SN*” (*n* = 64, no reported prior COVID-19, but lacked confirmatory test). Baseline samples from seropositive participants (group SP, *n* = 22) were also analyzed. For the infection-only samples (group I, *n* = 27), data were included if blood was collected from participants between 7 and 45 days following a PCR-positive nasal swab, with later samples collected 45–90 days post-infection designated “I45” (*n* = 29) and 90–135 days post-infection designated “I90” (*n* = 13); the infection-only group included participants who may have been seronegative or seropositive at baseline, but refers to samples collected after a PCR-confirmed infection detected on-study. Vaccine-only samples (group V, *n* = 75) included blood collected 7 to 45 days following the second mRNA vaccination from persons confirmed seronegative at enrollment. Samples collected from individuals 45–90 and 90–135 days post-second vaccination were designated “V45” (*n* = 66) and “V90” (*n* = 12), respectively. Samples collected 7 to 45 days following a second mRNA vaccination from participants who were seropositive at the time of vaccination reflect the development of hybrid immunity (group H, *n* = 19), with samples collected 45–90 days post-vaccination designated “H45” (*n* = 17) and 90–135 days post-vaccination designated “H90” (*n* = 5). A small subset of individuals in the study experienced a “breakthrough infection” after vaccination; we analyzed pre-breakthrough samples (group VPreB, *n* = 12) collected 7 days after full two-dose vaccination but before infection occurred, as well as post-breakthrough samples collected 7–45 days after infection (group VPstB, *n* = 12). Variation in the timing of sample collection relative to exposure events is shown in **Figure 1B**.

### 3.2 Serum and remnant material can be used to simultaneously profile neutralizing antibody and cellular immune responses

Participants provided whole-blood samples for SARS-CoV-2 clinical serology. Immediately after collection, the blood was coagulated at room temperature and subsequently centrifuged to separate clotted cells from the overlying serum for the purpose of anti-SARS-CoV-2 pseudo-neutralization assays. The remnant pelleted clot material (hereafter “clot”) was retained and stored at –80°C. To examine whether this remnant material could be used for profiling cellular immunity, we developed a new protocol for extraction of gDNA from the cells in the clot that was compatible with sequencing of the TCR repertoire (TCR beta-chain sequencing using Adaptive ImmunoSeq v4b assay, see Materials and Methods).

To assess whether we could gather sufficient information from TCRseq following our recovery protocol on recalcitrant clot material, we first quantified the number of unique productive rearrangements (UPRs) recovered from four control patient samples where both EDTA-stored whole blood and recalcitrant post-centrifugation serum clot material were available. Across these controls, the serum clot (SST) extraction yielded a comparable quantity of UPRs to traditional DNA extraction from whole blood, with yields ranging from −4.3% to +16% relative to the EDTA-blood comparator. For instance, in Participant 2438, the triplicate-pooled SST method yielded 452,645 unique clones compared to 396,310 clones from triplicate-pooled PBMC. Similarly, in Participant 3676, the SST method recovered 225,357 unique clones versus 194,956 from PBMC. Absolute UPR recovery naturally varies by individual (e.g., age, CMV serostatus, and recent infections), technical factors including the quantity of total input material, and DNA sequencing depth. The comparisons across four individuals demonstrate that the SST method captures an immune repertoire snapshot with diversity levels equivalent to, and in some cases exceeding, that of standard PBMC isolation. Next, we examined the frequency estimates of clonotypes co-detected by both methods (**Supplementary Figure 1A**). The concordance in clonal relative abundance between sample types mirrored the variance profiles typically observed in technical replicates of TCR repertoires. Highly abundant TCRs showed close agreement in frequency, while lower-abundance TCRs exhibited expectedly higher variability. We also did not observe strong systematic bias in frequency estimates; differential abundance analysis revealed almost no clones with statistically significant changes in relative frequency between the two methods (**Supplementary Figures 1B–E**).

Across the more than 450 TCRseq repertoires analyzed in the present CoVPN 3006 study, the recovery of TCR sequences from participants’ serum clot samples was robust, with UPRs ranging from 284,421 to 1,239,320 [interquartile range (IQR) 482,108–715,770]. This level of repertoire diversity recovered is comparable to previous large cohort TCRseq studies utilizing peripheral blood or PBMC (20, 21). We next considered how well these data were amenable to classification of SARS-CoV-2 exposure status using a TCRseq-based exposure classifier previously developed and validated by Adaptive Biotechnologies (22). Applying the previously defined classifier, nearly all pre−exposure repertoires from seronegative individuals were called negative (97.8%, 133/136). Likewise, 98.6% (73/74) of samples collected within 45 days of vaccination and 89.3% (25/28) of samples collected within 45 days of infection were called positive. The high diversity of repertoires and high accuracy of these classifier results demonstrate that remnant clot material could be reliably used after coagulation, serum recovery, and freezing for deep cellular immunological profiling. This was critical in the present study because participant PBMCs were not routinely collected, precluding the use of any live-cell T-cell assays.

### 3.3 Route of exposure is associated with neutralization potency

**Figure 2:**
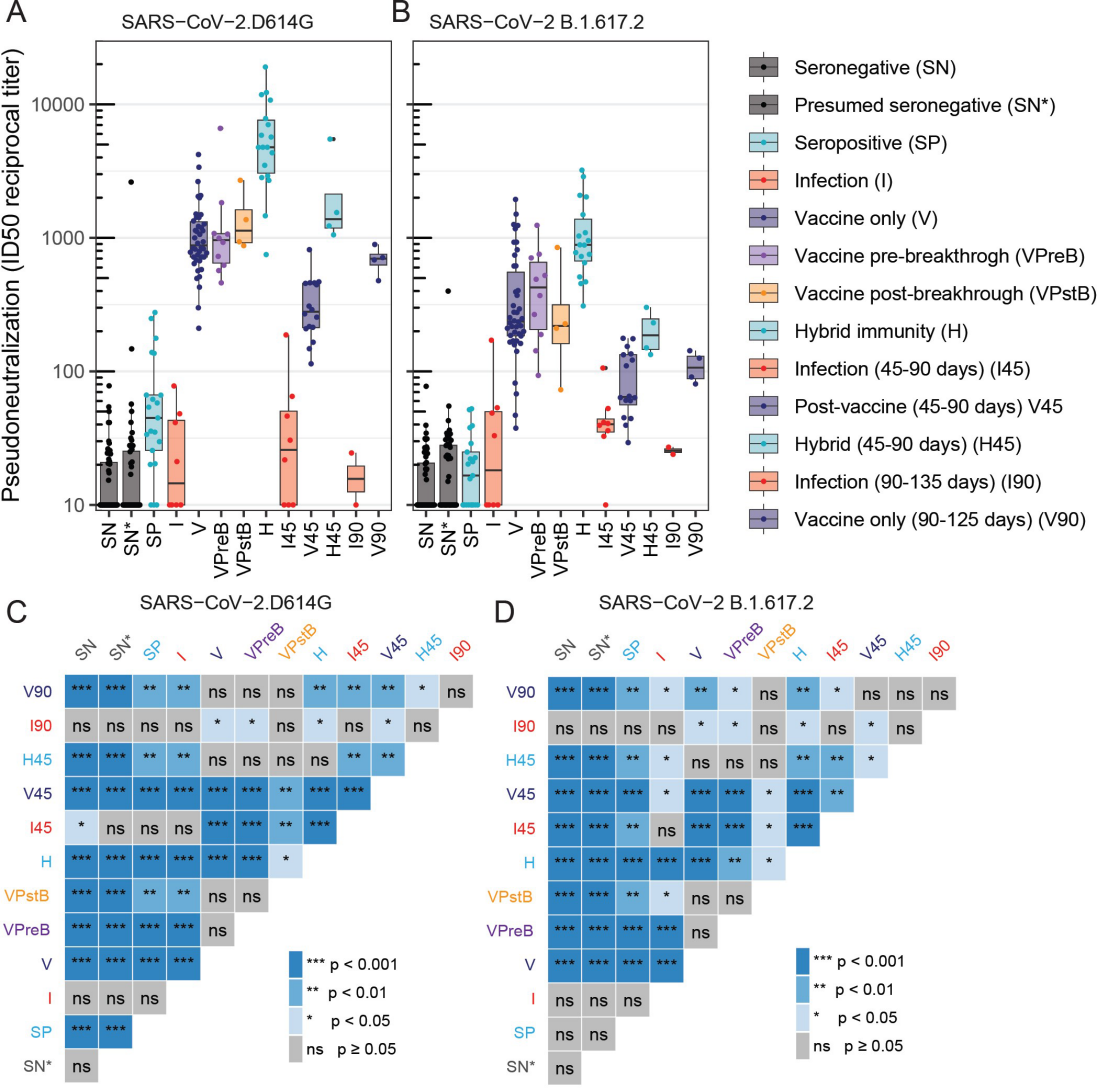
Pseudo-neutralization potential of serum from samples after a last known exposure. Samples were collected 7-45, 45-90 (+45), or 90-135 (+90) days after a last known exposure. Neutralization with a lentiviral pseudovirus-based assay against variants D614G **(A)** and B.1.617.2 (Delta) **(B)** are reported as reciprocal 50% inhibitory dilution (ID50) with a value of 20 (minimum dilution tested) plotted for samples that did not neutralize. Higher values indicate greater antibody potency. ID50 titers < 20 are plotted as half the limit of detection (10). Statistical significance was assessed using a two-sided Wilcoxon rank sum test with comparison between groups shown for neutralization titers against D614G **(C)** and B.1.617.2 **(D)**. P-values shown are not adjusted for multiple hypothesis tests. (*** *P* < 0.001, ** *P* < 0.01, * *P* < 0.05; ns, not significant).

To investigate if the mechanisms of SARS-CoV-2 exposure (infection, vaccination, or both) resulted in differences in the neutralization potency of serum, we compared the pseudo-neutralization potential of serum from samples collected 7–45 days, 45–90 days (+45), or 90–135 days (+90) after a last known exposure against five pseudo-virus variants: D614G, B.1.617.2 (Delta), B1.1.529 (Omicron), BA.4/BA.5 (late Omicron), and BQ1.1 (BA.5 subvariant) (**Figure 2**, **Supplementary Figure 2**, **Supplementary Table 2**, **Supplementary Materials**). All *p*-values and statistics can be found in the **Supplementary Materials**.

We measured the serum ID_50_ against each variant, which refers to the dilution of serum at which 50% neutralization is achieved, with higher values indicating greater antibody potency. For the variant closest to the vaccine immunogen (D614G), serum from baseline seronegative participants (SN) had a median ID_50_ titer of 10. Comparatively, serum from baseline seropositive participants (SP) had a median ID_50_ titer of 44.8, four times greater than SN serum samples, suggesting the presence of pre-existing spike-specific antibodies. Similarly, serum collected from participants following SARS-CoV-2 infection (I), had a median ID_50_ of 15.56. Of note, for the D614G variant, median ID_50_ of serum collected after vaccination (V, median = 877) showed 56 times greater potency than serum collected after infection alone (V *vs*. I, *p* < 0.0001) and was 20 times higher than serum collected from seropositive participants (V *vs*. SP, *p* < 0.0001). This relationship held for the variant closest to the circulating Delta strain (B.1.617.2), for which the serum from the vaccine group had 11 times greater potency than that from the infection-only group (I; *p* < 0.0001). For all pseudo-variants tested, serum ID_50_ was highest in samples collected after vaccination among participants with hybrid immunity (i.e., group I < V < H and I45 < V45 < H45) (**Figure 2**).

For D614G, median hybrid serum ID_50_ was 307 times greater than in serum collected after infection alone (*p* < 0.0001) and 5 times greater than vaccination alone (*p* < 0.0001). As expected, the assays showed that serum following vaccination had the highest neutralization potency against the vaccine matched pseudo-virus D614G, though participants with hybrid immunity (H and H_+45_) had potent neutralization against the non-vaccine-matched variants as well (ID_50_ > 100).

We also examined neutralization ID_50_ from samples collected further from the time of last exposure. Neutralization potency waned among all groups over 45–90 days (I45 < I, V45 < V, and H45 < H) and further waned for the samples collected 90–135 days post-exposure (**Figure 2**). Importantly, neutralization among the vaccine-alone and hybrid exposure group remained higher, even at later timepoints, compared to potency in the infection-alone group, suggesting greater durability of responses from vaccination. Altogether, these data show that exposure via vaccination (either alone or following natural infection) provide the most durable neutralization potency.

### 3.4 Vaccination against SARS-CoV-2 resulted in increased breadth of spike-specific T-cell receptors

**Figure 3:**
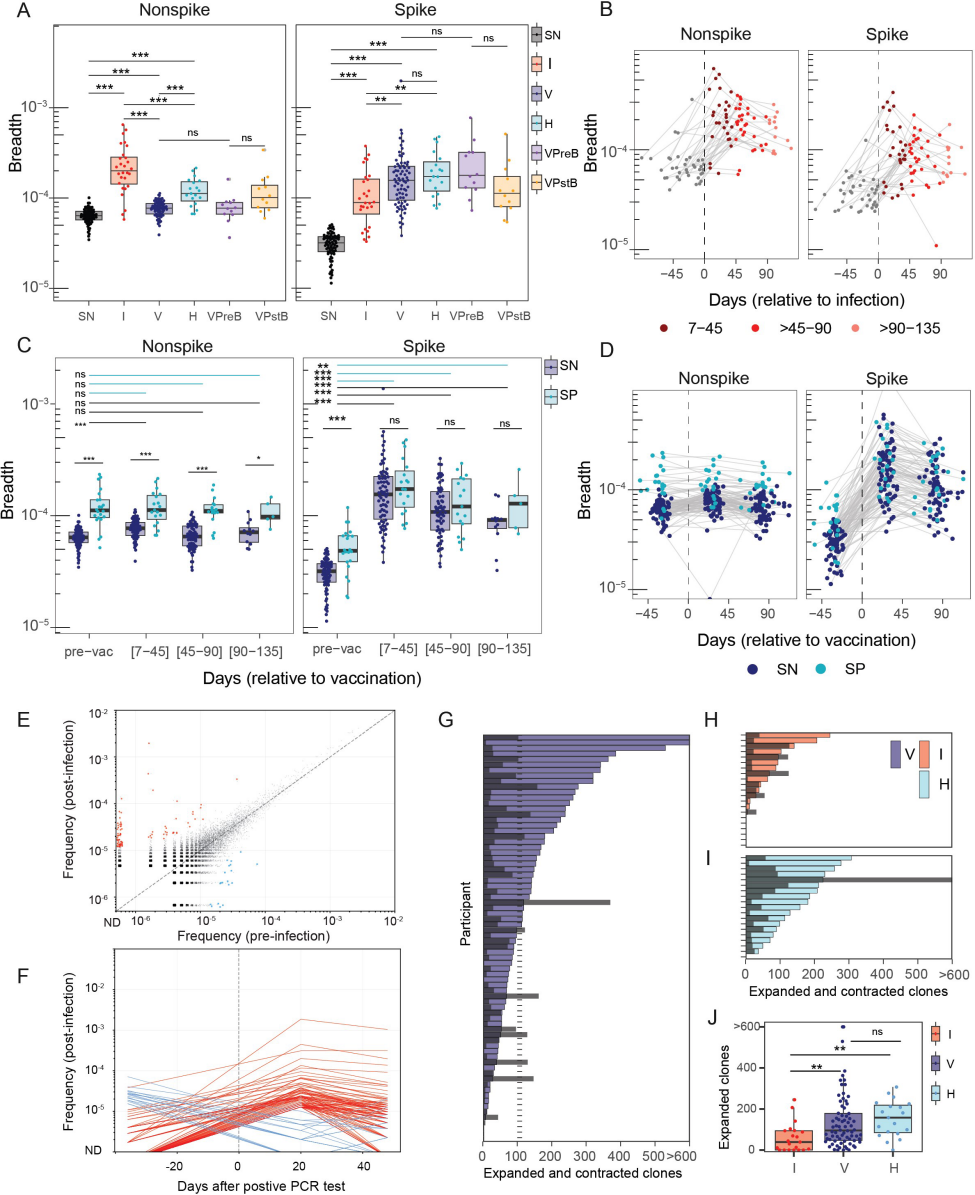
Spike- and non-spike-reactive T cell receptor beta-chain (TRB) sequences by exposure category. **(A)** T cell breadth (defined as the proportion of unique TCRs matching the database of public SARS-CoV-2 Spike and non-spike sequences) stratified by exposure status. Statistical significance was determined using a two-sided Wilcoxon rank sum test. **(B)** Longitudinal T cell breadth in unvaccinated participants before and after confirmed SARS-CoV-2 infection. Gray lines connect samples from the same participant. **(C)** T cell response breadth for seronegative (SN, black) and seropositive (SP, cyan) individuals at intervals relative to the second mRNA vaccine dose. Significance was calculated using a two-sided Wilcoxon rank sum test. **(D)** Longitudinal T cell breadth in seropositive and seronegative participants before and after a two-dose mRNA vaccination series. Gray lines connect samples from the same participant. **(E)** Scatter plot of clonal frequencies pre- versus post-infection in a representative participant. Colored points indicate significantly expanded (red) and contracted (brown) clones (fold change > 4; FDR < 0.05). Analysis is restricted to clones with ≥5 copies at either timepoint. A slight jitter was applied to minimize overplotting. **(F)** Longitudinal trajectories of the expanded and contracted clones identified in **(E)**. **(G–I)** Number of expanded clones per participant following vaccination in seronegative individuals **(G)**, following infection **(H)**, and following vaccination in seropositive individuals **(I)**. Gray bars indicate the number of contracted clones. **(J)** Number of expanded clones stratified by exposure status. Significance was determined by two-sided Wilcoxon rank sum tests (*** *P* < 0.001, ** *P* < 0.01, * *P* < 0.05; ns, not significant).

To evaluate cellular immune responses among study participants, we generated TCR beta-chain repertoires from samples collected before and after exposure events. The breadth of spike (S) and non-spike (NS) specific receptors were quantified from each TCR repertoire using a database of public TCR sequences developed by Adaptive Biotechnologies (23); breadth is the proportion of unique TCRs that match this database. We observed that the breadth of S-specific TCRs was higher after vaccination compared to after infection alone (**Figures 3A, B**, **Supplementary Materials**; *p* = 0.008). The breadth of NS-specific TCRs differed before and after vaccination between vaccinated participants who were seropositive *vs*. seronegative at the time of the first vaccine (**Figures 3C, D**). Participants with hybrid exposure had increased non-spike TCRs compared to the vaccine-alone (V) groups (*p* < 0.0001, **Supplementary Materials**), which reflects exposure to non-spike antigens during infection. Notably, there was not a significant difference in the breadth of S-specific TCRs among participants in the hybrid (H) compared to the vaccine-only (V) group, despite the additional exposure in the hybrid group (**Figure 3A**, **Supplementary Materials**; *p* = 0.26). The higher TCR breadth observed in post-vaccination compared with post-infection samples was mirrored by the magnitude of changes we observed in TCR repertoire when considering the number of clones that substantially changed in abundance between pre- and post-exposure timepoints (**Figures 3E, F**). We defined TCR receptor sequences as an expanded or contracted clonotype if it expanded in frequency by more than 4 times or contracted by more than 0.25 and passed a Fisher’s exact test with an FDR-adjusted *p* < 0.05 (24). Across post-infection and post-vaccination samples, we observed more expanded than contracted clones (**Figures 3G–I**). Post-vaccine samples had more expanded clones compared with post-infection samples (**Figures 3H, J**, *P* = 0.002) with 6 of 21 post-infection samples having zero expanded clones relative to the pre-infection timepoint. After 2 dose vaccination, we did not observe a statistically significant difference in the number of expanded clones between seropositive (H) and seronegative (V) recipients of the vaccine (**Figures 3H, J**, *P* = 0.19) consistent with similar levels of S TCR breadth measured between these groups after vaccination (**Figure 3A**). We mapped statistically significant expanded sequences using TCRdist-KNN to sets of class I MHC S peptide-associated TCRs derived from prior MIRA assays and experimental screens (25, 26), with polyclonal expansions co-mapping to previously identified immunodominant peptides [e.g., S_269–277_ (A*02:01 YLQPRTFLL), S_378–385_ (A*03:01 KCYGVSPTKL), S_448-458_ (A*24:02 NYNYLYRLF), and S_448-458_ (B*15:01 NQKLIANQF), **Supplementary Figure 4**].

Among participants in the infection-only, vaccine-only, and hybrid groups, S-specific TCR breadth was lower in samples collected +45 and +90 days after the last known exposure compared with sample collected 7 to 45 days after exposure (**Figure 3B**, **Supplementary Materials**). However, despite the decrease in S-specific TCR breadth with time, the later vaccine and hybrid exposure samples retained greater S-specific TCR breadth at all timepoints compared to samples collected post-infection. The lack of samples from later timepoints precluded an evaluation of differential durability; however, these results suggest that exposure to SARS-CoV-2 spike via vaccination led to increased cellular recognition compared to infection alone.

### 3.5 Order of exposure to SARS-CoV-2 impacts neutralization potency

For the participants who experienced a breakthrough infection after vaccination (*n* = 12), neutralizing antibody acquired following infection was not greater than those following vaccination only. It was notable that neither neutralization potency (against any SARS-CoV-2 variant) nor nonspike- and spike-specific TCR breadth were substantially increased following a breakthrough infection, compared to pre-breakthrough levels (*p* > 0.05 for all variants, *p* = 0.060 for S and NS; **Figures 2**, **3**, **Supplementary Figures 2**, **3**, **Supplementary Materials**).

We also compared the immune responses after breakthrough infection (vaccination followed by infection) with those of participants who experienced a hybrid exposure (infection followed by vaccination) to interrogate how the order of exposure affected immunity. Hybrid immunity resulted in much greater neutralization potency than breakthrough infection for all the variants tested (**Figure 2**, **Supplementary Figure 2**). For example, samples collected from individuals with hybrid immunity had four times higher neutralization potency against the D614G variant (median = 4,771) compared to those from individuals who experienced a breakthrough infection (median = 1,152) (VPstB < H, *p* = 0.0074, **Supplementary Figure 2** and **Supplementary Materials**). The median breadth of S-specific TCRs from participants with hybrid exposure (median = 0.00017) was also greater than the median breadth from participants who experienced breakthrough infections (median = 0.00011). However, this did not reach statistical significance, *p* = 0.10 (**Supplementary Materials**); this observed trend was also similar for non-spike TCR breadth (**Supplementary Materials**).

We hypothesized that individuals with a breakthrough infection may have had lower antibody and/or T-cell responses prior to the infection (VPreB) compared to other participants who did not experience breakthrough (V). There was no significant evidence that individuals having a subsequent breakthrough infection had lower neutralization potency (**Figure 2**; *p* > 0.05, **Supplementary Materials**) across all variants tested (e.g., D614G ID_50_ 960 *vs*. 877 for VPreB *vs*. V, *p* = 0.864). Spike and non-spike TCR breadth were also comparable for individuals with breakthrough infection compared to all other vaccinated individuals (**Figure 3**; *p* > 0.05 for both, **Supplementary Materials**).

### 3.6 Vaccination attenuates duration of infection and viral load

To better understand the relationship between immunity and infection, we investigated high-resolution longitudinal viral shedding data from daily swabs provided by participants. Participants performed self-swabs daily, starting at vaccination and continuing for 16 weeks. They were instructed to return samples to sites three times a week. A positive PCR result triggered the testing of samples from 3 days prior to 14 days after the positive specimen or until viral RNA as assessed by PCR was no longer detected. More than 122,000 swab samples were collected in the main study (8).

**Figure 4:**
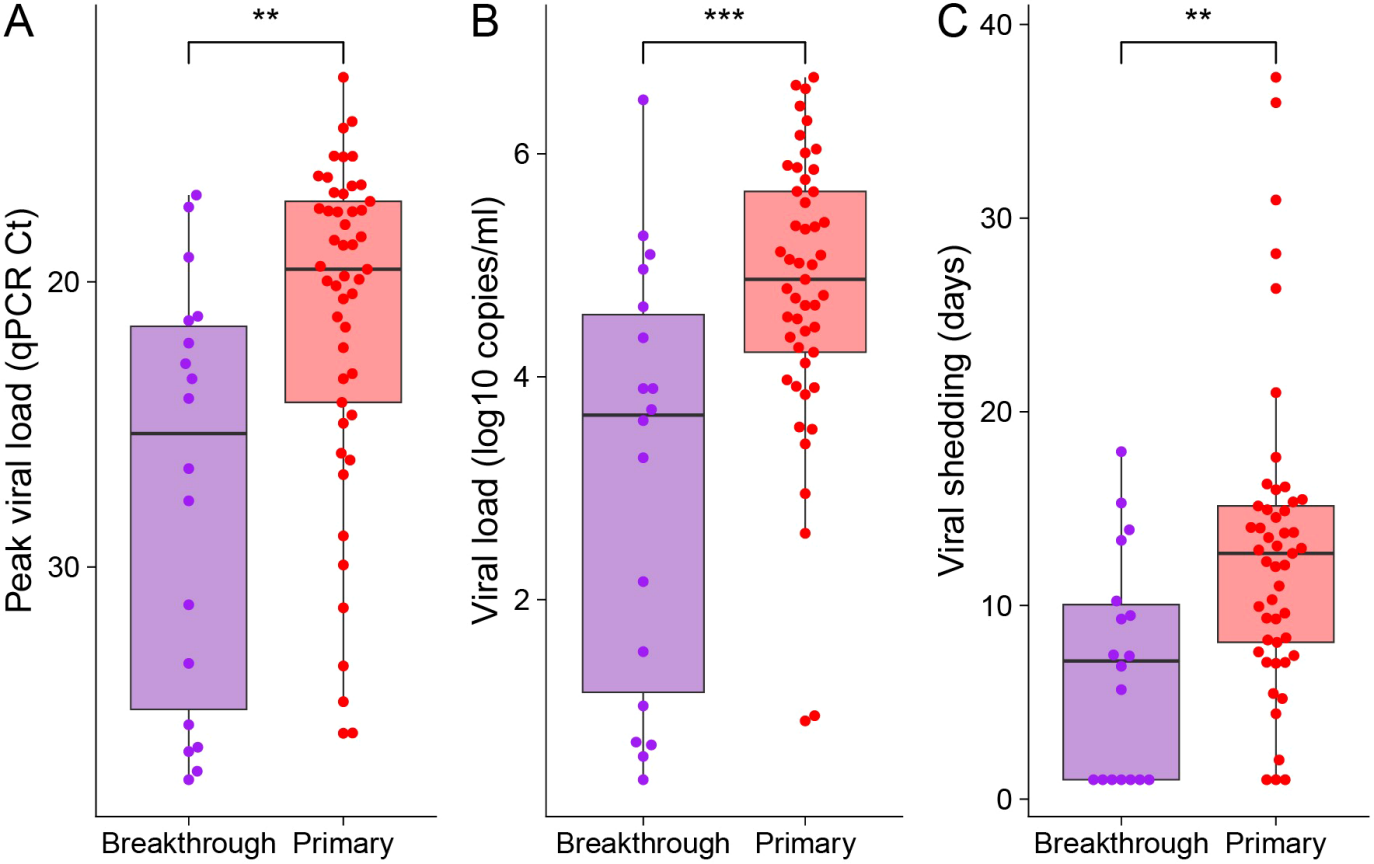
Viral load and viral shedding in breakthrough and infection-only samples. **(A)** Peak viral load inferred from qPCR critical threshold (Ct). **(B)** Peak viral loads and **(C)** viral shedding durations estimated using a Bayesian hierarchical model. Participants were instructed to perform self-swabs on the anterior nares daily starting at vaccination or day 1 (depending on group) and continuing for 16 weeks. To determine peak viral load and viral shedding duration, positive PCR result triggered testing of swab samples from 3 days before to 14 days after the positive specimen or until viral RNA was no longer detected. Estimated viral shedding duration after first PCR positive daily swab. ** *p* < 0.01 *** *p* < 0.001.

We found evidence of greater immunological control in breakthrough infection compared to infection-only (**Figure 4**). We analyzed the critical threshold (Ct) from the qPCR assays targeting loci on the SARS-CoV-2 nucleocapsid gene, with lower values indicating higher starting template concentration associated with higher viral burden. The minimum Ct in samples following primary infection was 19.5 [IQR 17.2-24.2] compared with 25.3 [IQR 21.5–35] following breakthrough (**Figure 4A**, *p* = 0.0016). We also estimated peak viral load from all swabs using a Bayesian hierarchical model (see Materials and Methods); the median viral load following primary infection was 4.9 [IQR 4.2–5.7] compared with 3.6 [IQR 1.2–4.5] following a breakthrough infection (**Figure 4B**, *p* = 0.0005). Median total shedding duration estimated by Bayesian hierarchical model for primary infection was 12 [IQR 9–15] compared with 7 [IQR 1–10] days for breakthrough infection (**Figure 4C**, *p* = 0.003). We further examined the degree to which participants reported symptoms associated with a positive PCR result. Among breakthrough cases, we did not detect an association between duration of viral shedding and presence of symptoms. However, in primary infections, symptomatic cases were associated with longer period of viral shedding (**Supplementary Figure 5**). The duration of viral shedding observed in breakthrough infections more closely resembled shedding duration in asymptomatic primary cases (**Supplementary Figure 5**). Together, these results suggest that vaccination improved immunological control of subsequent infections.

## 4 Discussion

We characterized SARS-CoV-2 immunity before and after mild SARS-CoV-2 infection and mRNA-1273 vaccination, using frequent swabbing to rapidly identify participants after infection. The unique study design and development of a novel protocol to simultaneously profile serum neutralization and TCR repertoires from a single sample enabled direct comparisons of immune responses before and after heterogeneous SARS-CoV-2 exposures in 209 participants. We demonstrated that the mechanism and order of exposure to SARS-CoV-2 affected both cellular responses and neutralizing antibody levels, as well as viral burden in breakthrough infection. While many of the observations in this study validate the observations of previous studies, for example, the robust immunity derived from mRNA vaccination (27, 28), the superior immunity from hybrid exposure (29, 30), and the resulting attenuation of breakthrough infection (29, 30), here the heterogeneity of exposures in a single clinical trial, the longitudinal sampling and focus on mild and asymptomatic infection, extends the published literature. In most participants, mRNA vaccination elicited strong antibody and T-cell responses to the vaccine-matched SARS-CoV-2 spike protein. This is consistent with previous studies that have characterized vaccine-induced increases in serum neutralization and its association with a lower risk of COVID-19 (31). Thus, we hypothesized that post-vaccine neutralization potency would be lower among participants who eventually experienced a breakthrough infection, compared to other vaccine recipients who did not. Instead, we observed that prior to a breakthrough infection, participants had similar or only an incremental increase in neutralization potency compared to the vaccine-only group.

Importantly, in breakthrough infections following vaccination with a heterologous ancestral mRNA vaccine, we observed reduced viral shedding duration and lower peak nasal viral load, compared to naïve infections. Although disease was mild or asymptomatic among all participants, this demonstrated the impact of vaccine-induced immunity on the virus. Notably, the reduced viral exposure also reduced the subsequent immune response to such an extent that spike TCR breadth and neutralization potency were not boosted and/or continued with contraction (TCR breadth).

In contrast to muted immunological responses in breakthrough infections, we observed that mRNA vaccination of seropositive individuals resulted in strong boosting of neutralization potency beyond what was observed for either vaccination or infection alone. In this group with so-called “hybrid immunity”, vaccination after a prior infection boosted anti-S antibodies to levels sufficient for neutralization of multiple SARS-CoV-2 pseudo-viral variants including those with Omicron-type spike proteins. Moreover, the hybrid group had reduced viral shedding in breakthrough infection, highlighting the importance of vaccination even after natural exposure to SARS-CoV-2. This study is one of the first to evaluate the development of anti-SARS immunity in young adults and reinforces prior studies (29, 30, 32), showing that the timing of repeated SARS-CoV-2 antigen exposure, involving combinations of vaccination and infection, can alter the magnitude of immunological responses.

A limitation of this study is the lack of a comprehensive comparison of TCR repertoires amplified from blood clots versus paired whole blood or PBMC samples. Consequently, there exists the possibility of subtle technical biases inherent to the clot-based extraction protocol. This method was specifically designed to address a critical gap in large-scale vaccine studies where live-cell preservation is not routine. Despite the absence of a direct whole blood comparator for CoVPN3006 clot-derived TCR repertoires, the recovered repertoire data were diverse, successfully enabled longitudinal clonal tracking, and captured clear signals of antigen exposure. Thus, while sample type-specific nuances may exist, this protocol unlocks the potential for immunogenomic analysis in cohorts where only archived frozen blood clots are available.

Another limitation of this study was the imperfect compliance with daily nasal swabbing, as a result of the trial design. This reduced precision for measuring peak viral shedding and duration. Moreover, the small number of breakthrough infections was a limitation of this study, as was the complexity of sample collection timing. Although we attempted to categorize the samples in meaningfully narrow ways, the timing of samples was not always connected to the exposure event, which required sample exclusions. Although the consistently mild severity of disease supported the importance of vaccination, the absence of severe disease in the cohort and the small size of the breakthrough infection cohort precluded our ability to correlate viral shedding with immune response. Finally, it is important to note that comparisons of immune markers between groups were not adjusted for participant features that could possibly have confounded the results. Though vaccination was randomized, comparisons focusing on subgroups defined by baseline serostatus were exploratory and based on limited blood samples and data that were available for analysis. The results are descriptive and leverage non-parametric statistics that require minimal distributional assumptions; however, further experiments and sufficiently powered randomized comparisons would be required for mechanistic or causal interpretation.

Despite waning levels of neutralizing antibody responses and the emergence of variants that escape neutralization, there remain low levels of severe COVID-19 among vaccinated individuals globally (33). Many scientists speculate that cellular immunity provides protection against severe disease, when neutralizing antibody fails to prevent infection (34–36). However, there is little direct evidence linking T-cell responses to reduced disease severity. The sparsity of evidence is partly due to the challenges of quantifying spike-specific T-cell responses from large, vaccinated cohorts using gold-standard methods like enzyme-linked immunosorbent spot (ELISpot) and flow cytometry, which require viable cryopreserved PBMCs. Here, we show the potential value of quantifying spike and non-spike T cells from the remnant material of processing serum from whole blood. While the catalog of TCRs is not yet comprehensive, we show that the breadth of TCRs in an individual’s repertoire is reliably expanded by antigen exposure through infection and vaccination. Though TCR breadth is not a substitute for functional T-cell readouts, it may be a more accessible and standardized biomarker of cellular immunity that can be used to assess the role of T cells as correlates of protection in large clinical trials.

## Data Availability

The original contributions presented in the study are publicly available. This data can be found here: doi.org/10.6084/m9.figshare.31807288.
