# Supplementary material for: Profiling of SARS-CoV-2 virus shedding, antibody neutralization, and T-cell receptor repertoires in a large, multi-center cohort of young adults with varied prior exposures
Source: Front Immunol. 2026 Mar 25;17:1731974. doi: 10.3389/fimmu.2026.1731974 (PMC13057264; doi:10.3389/fimmu.2026.1731974)
Supplement: Supplementary file 1 [file DataSheet1.pdf]

Supplementary Material

1 Supplementary Figures and Tables

1.1 Supplementary Figures

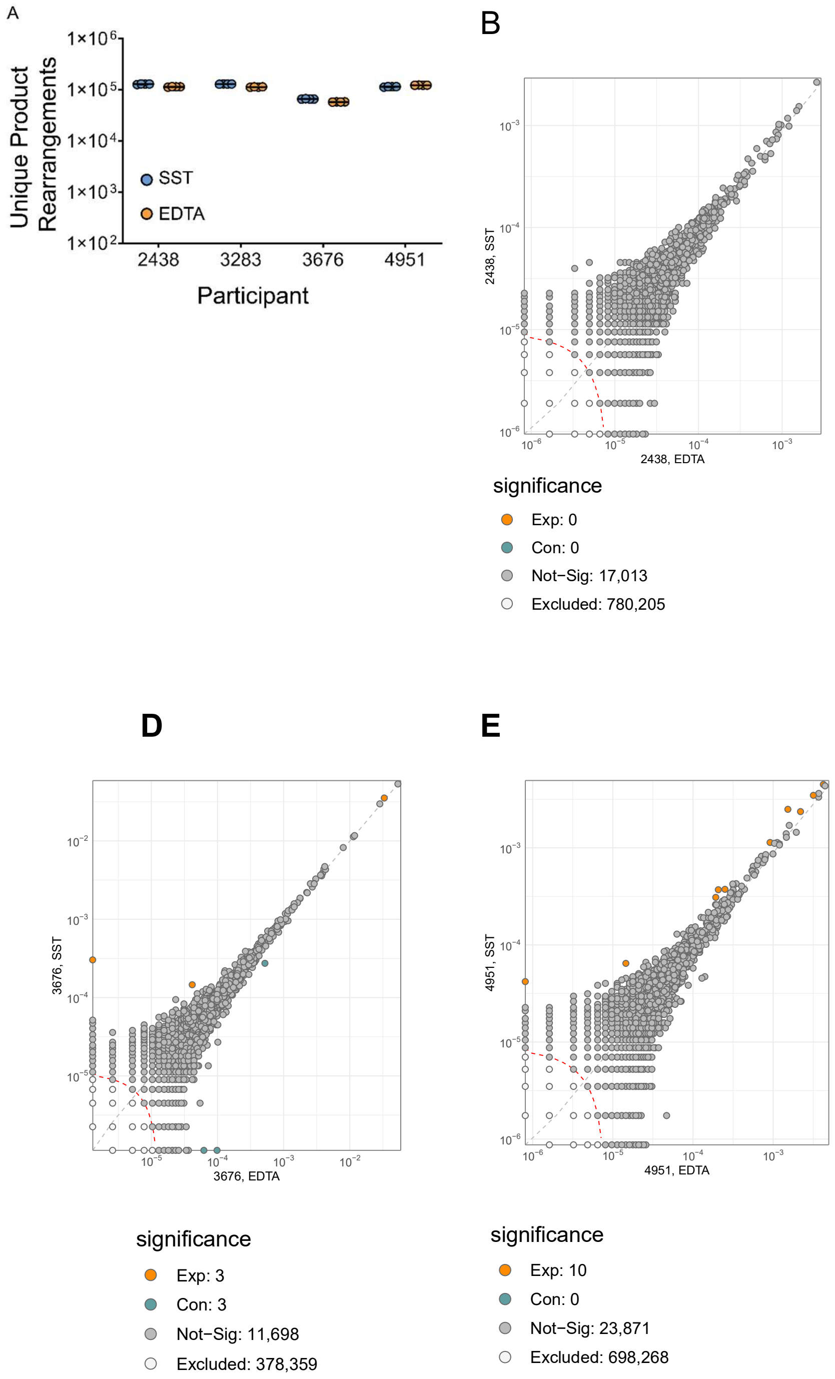

Supplementary Figure 1: Profile of TCR repertoire from SST clotted blood and EDTA blood. (A) Estimate of productive T cell receptor sequences detected in donor-matched genomic DNA isolated from four participant samples prepared using SST frozen clotted blood or frozen EDTA preserved blood. (B) – (E) Differential clonal abundance in serum separator tube (SST) clotted blood and EDTA-stored blood from each participant: 2438, 3283, 3676, and 4951. Exp: Expanded; Con: Contracted.

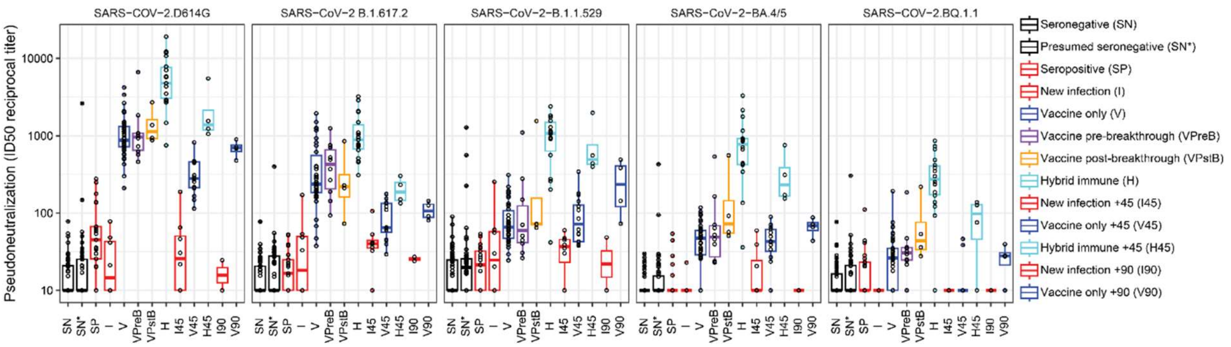

Supplementary Figure 2: Pseudo-neutralization potential of serum from samples after a last known exposure against pseudo-virus variants D614G, B.1.617.2 (delta), B.1.1.529 (omicron), BA.4/5 (late omicron), and BQ1.1 (BA.5 variant). Samples were collected 7-45, 45-90 (+45), or 90-135 (+90) days after a last known exposure. ID50 refers to the dilution of serum at which 50% of the pseudo-virus is neutralized, with higher values indicating greater antibody potency. Neutralization with a lentiviral pseudovirus-based assay and reported as reciprocal 50% inhibitory dilution (ID50) with a value of 20 (minimum dilution tested) plotted for samples that did not neutralize. Higher values indicating greater antibody potency. See Supporting Information for p values.

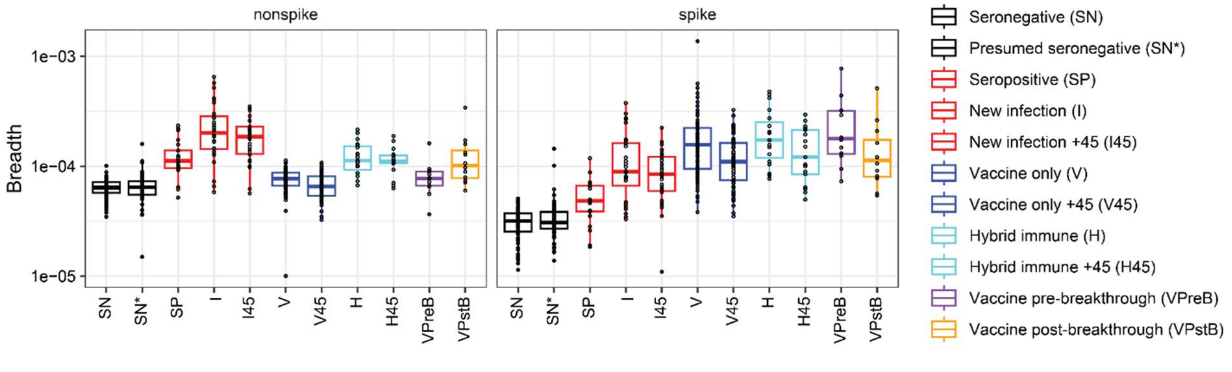

Supplementary Figure 3: (A) non-spike and (B) spike reactive T cell receptor beta-chain (TRB) sequences by exposure category. Colors are used to indicate different exposures: baseline (black), infection and later timepoints (red), vaccination and later timepoints (blue), hybrid immunity and later timepoints (cyan), and pre and post breakthrough (purple and orange, respectively). Early groups (I, V, H) refer to 7-45 days post more recent exposure, and +45 (45-90 days), and +90 (90-

135 days). The level of statistical significance calculated using a two-sided Wilcoxon rank sum test. See Supporting Information for p values.

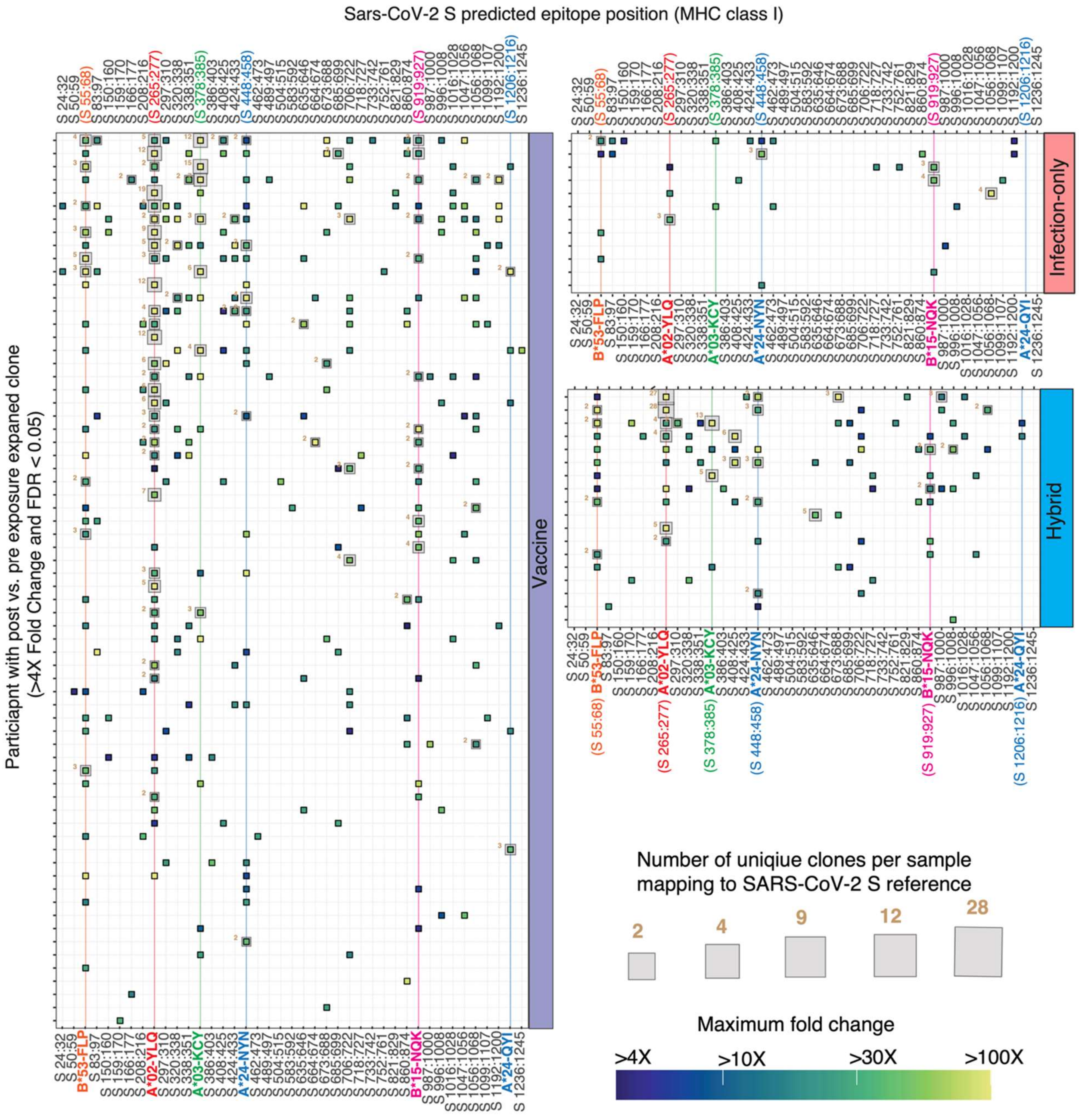

Supplementary Figure 4: Clones that substantially (fold change > 4) and significantly (FDR < 0.05) expanded after exposures were mapped to a reference set of TCRs experimentally defined to recognize SARS-CoV-2 Spike peptides based on prior studies (Nolan et al. 2025; Minervina et al. 2022; Ford et al. 2024). Clones with a TCRdist-KNN (k = 7) score > 0.8 to a single epitope reference set are shown. Each row represents a participant with paired pre/post repertoires and at least one mapped expanded clone. The number of unique expanded clones per participant mapping to a single antigen target is shown by the size of the gray boxes, with the color of the inner box indicating the maximum fold change observed for any clone assigned to that target. The positions of major immunodominant epitopes are indicated by colored vertical lines. Panels are split among vaccine-only exposure, infection-only exposure, or hybrid-immunity groups (vaccination of seropositive participants).

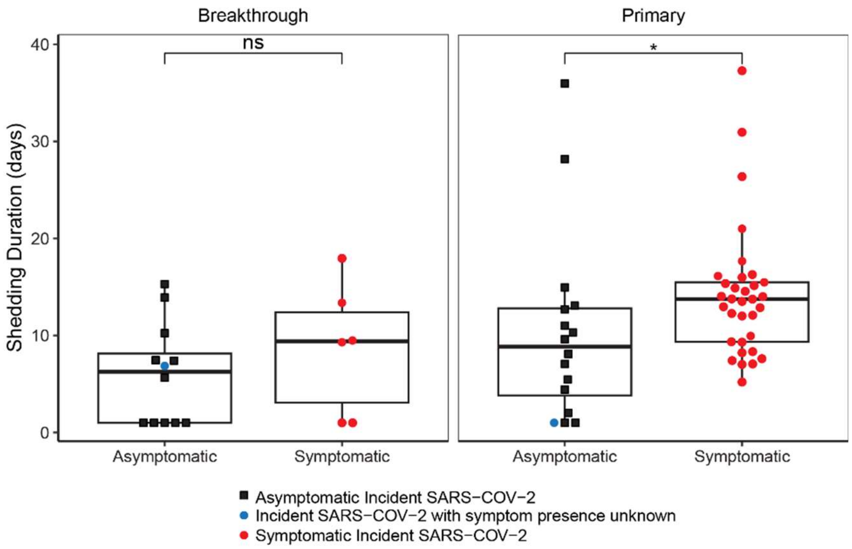

Supplementary Figure 5: Viral shedding in breakthrough and primary infection samples by **symptoms status.** Estimated viral shedding duration after first PCR positive daily swab. Participants were instructed to perform self-swabs on the anterior nares daily starting at vaccination or day 1 (depending on group) and continuing for 16 weeks. To determine viral shedding duration, positive PCR result triggered testing of swab samples from 3 days before to 14 days after the positive specimen or until viral RNA was no longer detected. * $P < 0.05$.

1.2 Supplementary Tables

Supplementary Table 1: Participant Demographics

		Overall	Infection/Hybrid	Reference	Vaccine
		N[1] = 202	N = 68	N = 39	N = 95
**Age**	Years	21.0 (18.0, 29.0)	21.0 (18.0, 29.0)	22.0 (18.0, 29.0)	21.0 (18.0, 29.0)
**Sex**	Female	127 (63%)	45 (66%)	24 (62%)	58 (61%)
	Male	75 (37%)	23 (34%)	15 (38%)	37 (39%)
**Race**					
	American Indian or Alaska Native	1 (0%)	0 (0%)	0 (0%)	1 (1%)
	Asian	20 (10%)	2 (3%)	2 (5%)	16 (17%)
	Black or African American	22 (11%)	11 (16%)	5 (13%)	6 (6%)
	Multiple	13 (6%)	5 (7%)	2 (5%)	6 (6%)
	Other	6 (3%)	2 (3%)	1 (3%)	3 (3%)
	Unknown	3 (1%)	1 (1%)	0 (0%)	2 (2%)
	White	137 (68%)	47 (69%)	29 (74%)	61 (64%)

[1] Median (Min, Max); n (%).
Infection/Hybrid group includes participants with last sample in exposure categories SP, I, I45, I90, H, H45, or H90.
Vaccine group includes participants with last sample in exposure categories V, V45, V90, VPreB, and VPstB.
Reference group includes participants with in exposure category SN*.

Supplementary Table 2. Median pseudoneutralization in ID50 reciprocal titer against SARS-CoV-2 variants

	D614G	B.1.617.2	B1.1.529	BA.4/BA.5	BQ1.1
Seronegative (SN)	10	10	10	10	10
Presumed seronegative (SN*)	10	10	19.83	10	10
Seropositive (SP)	44.84	16.57	21.28	10	10
New infection (I)	15.56	21.50	25.12	10	10
Vaccine only (V)	877.78	236.95	65.66	46.96	26.18
Vaccine pre-breakthrough (VPreB)	960.36	429.72	60.39	48.88	30.56
Vaccine post-breakthrough (VPstB)	1152.40	218.82	72.01	74.26	44.72
Hybrid immune (H)	4771.37	884.91	1073.51	775.35	275.42
New infection +45 (I45)	26.14	40.10	36.89	10	10
Vaccine only +45 (V45)	279.91	64.40	72.82	422.64	10
Hybrid immune +45 (H45)	1388.85	190.71	496.46	241.07	101.11
New infection +90 (I90)	17.25	25.50	29.16	10	10
Vaccine only +90 (V90)	698.27	108.26	262.88	68.54	27.70
